# The influence of service quality and anticipated emotions on donor loyalty: an empirical analysis in blood centres in Spain

**DOI:** 10.1007/s10729-022-09600-9

**Published:** 2022-07-16

**Authors:** Josefa D. Martín-Santana, Lucía Melián-Alzola

**Affiliations:** Universidad de Las Palmas de Gran Canaria, Campus de Tafira s/n, 35017 Las Palmas de Gran Canaria, Las Palmas Spain

**Keywords:** Blood donation, Service quality, Anticipated emotions, Loyalty, structural equation models (SEM)

## Abstract

Blood donation centres need to recruit and retain donors to ensure the effectiveness and efficiency of healthcare systems, as COVID-19 has recently evidenced. In such risky settings, blood donation services must increase donations. Service quality can increase donations but its evaluation only amounts to a cognitive evaluation, and not to an emotional appraisal. Consequently, both service quality and emotions should be considered when predicting donor behaviour. In fact, donating blood is an emotionally charged service, thus representing an ideal setting to investigate how emotions influence consumer behaviour. This research proposes a new method to predict blood donors’ intentions by integrating a cognitive approach measuring perceived quality, and an emotional approach including anticipated emotions (both positive and negative) of ‘donation’ and ‘non-donation’. Based on a sample of 30,621 active Spanish donors, it is concluded that service quality is an antecedent for anticipated emotions and that both service quality and anticipated emotions influence donor loyalty. Designing the donation process based on quality criteria would provoke encouraging emotions and diminish discouraging emotions, therefore improving donor loyalty.

## **Highlights**

- Donating blood is a behaviour with a strong emotional charge.
- Anticipated emotions also play an important role in the donation decision.
- It is fundamental for blood transfusion centres to optimise their service processes.
- Service quality is an antecedent of anticipated emotions.
- Service quality and anticipated emotions influence donor loyalty.

## Introduction

The healthcare system needs to increase blood donations, in order to address the demand. Lacetera and Macis [1] state that only a small percentage of individuals eligible to donate blood (5%-10%) are actually blood donors in the Western world, reaching smaller percentages in developing countries. To et al. [2] have also pointed out that the blood supply has been decreasing more rapidly than the demand in US in recent years. The World Health Organization [3], with data from 2018, states that the number of donations per 1,000 inhabitants is 31.5 (high-income countries), 6.8 (middle-income countries) and 5.0 (low-income countries), with 72 countries reporting less than 10 donations per 1,000 inhabitants. These statistics indicate a significant opportunity for improvement. The management of blood donation centres should apply consumer behaviour models from the marketing and service management literature, although consumer behaviour is such a complex issue. Although the recruitment of new donors contributes to increasing the size of the donor pool and to replacing donors who, either voluntarily or obligatorily, cease to donate [4], retaining active donors implies lower costs [5]. Frequent donors reduce costs because they provide safe and sustainable blood supply [5]. Donation campaigns costs also would decrease as donating becomes habitual for experienced donors [6] and frequent donors have the advantage of knowing the donating process [7]. These data reveal that donor recruitment and, particularly, donor retention is very important for the sustainability of the health system. Some authors suggest that it is necessary to analyse and improve the perceived quality of the donation process in order to strengthen the donor’s intentions [e.g. 8, 9]. Nevertheless, this service quality evaluation amounts only to a cognitive evaluation, since the subject assesses the goodness/badness of the different service elements [10]. Literature suggest that it is also necessary to incorporate emotions, with the aim of considering feelings, in predictive models of consumer behaviour [e.g. 10–14].

Based on above, this study is firstly aimed at making an academic contribution to the literature on services by providing a theoretical and empirical basis for an area of research into blood donation where, despite its social importance, information is scarce. Secondly, this study integrates the cognitive approach measuring perceived quality and the emotional approach predicting the blood donor’s intentions. It delivers a holistic view to the traditional methods of consumer behaviour analyses found in the literature on service quality. More specifically, this research analyses the role of anticipated emotions (positive and negative) of ‘donation’ and ‘non-donation’ on donor loyalty, following Bagozzi et al. [15]. Finally, what should also be highlighted is the contribution this work makes to analysing the role of service quality as an antecedent of anticipated emotions. Although academic studies have been carried out to analyse the relationship between perceived quality and emotions in general [16], the role of perceived quality as a determining factor of anticipated emotions from two perspectives (donation versus non-donation) is a research area that is yet to be explored.

This paper analyses the simultaneous effect of service quality and anticipated emotions on donor loyalty. The framework to build the research model and analysis of service quality, anticipated emotions and donor loyalty, as with analysing and discussing the results of the research, is as follows. To analyse how perceived quality of the donation process influence both donor loyalty and anticipated emotions (positive and negative) of ‘donation’ and ‘non-donation’, as well as the effect of anticipated emotions on loyalty, the following section presents the theoretical basis for the hypotheses of the proposed model (Section 2). The study methodology (Section 3) and the research results (Section 4) are shown. The results are discussed, and the main conclusions of the study (Section 5) are drawn. These key findings revealed the positive influence of service quality and anticipated emotions on donor loyalty. Service quality positively influences the anticipated emotional response that motivates donation (positive anticipated emotion of donation and negative anticipated emotion of non-donation). Service quality has a negative impact on the emotional response that discourages willingness to donate (positive anticipated emotion of non-donation and negative anticipated emotion of donation). Regarding the influence of anticipated emotions on donor loyalty, both categories of anticipated emotions motivating donation enhance donor loyalty, as both categories of anticipated emotions motivating non-donation deter donor loyalty. Findings demonstrate that the degree of donor experience influences the relationship between negative anticipated emotions of donating and donor loyalty.

## Theory and hypotheses

### Blood donation in the service economy: A voluntary act

Donation behaviour is a unique type of exchange because a product or service is rarely received [17]. Whereas consumer choice depends on the perceived benefits a consumer receives from a monetary exchange, blood donation value concerns the perceived benefits a donor receives in return for a donation. Thus, individuals donate in order to receive something in return, such as status, recognition or emotional fulfilment [18]. Chen, et al. [19] also state that blood donation value is defined as the emotional experience value that blood donors obtain by saving others through the selfless contribution of blood donation. Chell and Mortimer [18] and Zainuddin and Gordon [20] continue to add that donation centres should maximise the value of the donor with particular focus on the donation experience. Thus, blood donation is both a cognitive and emotional process, as suggested by Williams et al. [21]. As Previte et al. [22] note, literature suggest emotions have also a central role to consumer experiences, particularly in non-commercially focused exchanges.

This paper aims to shed light on the literature by discussing how anticipated emotions affect the relationship between service quality (blood donation service) and customer (donor) loyalty. The conclusions drawn will provide a more in-depth understanding of the consumer behaviour when faced with a voluntary service, which is not a ‘need’ on the part of the consumer and does not generate an immediate tangible outcome, and where the outcome is mainly aimed at a third party.

### The donation process: An experience of quality service and emotions

Quality of service is an area of high academic and professional interest for healthcare institutions [23–25], among other reasons, because of increased focus on patient-centred care [25]. Donabedian’s structure-process-outcome approach model for measuring quality service in healthcare context [26] is widely recognised, in which structure enhances process, and process improves outcomes [24, 27, 28]. Upadhyai et al. [25] propose a multidimensional structure, based on literature review, whereby the dimensions of healthcare service quality can be categorised under medical and non-medical variables. Medical dimensions include technical (professional competence and medical facilities), outcome (effective and patient-centred care) and interpersonal (information exchange process) dimensions. Non-medical dimensions comprise servicescapes (basic amenities and physical environment), accessibility (cost, time and proximity), and responsiveness (expectations from care which is acceptable as a human being) dimensions.

Evaluating the quality of the health service from the customer's perspective requires measuring aspects that are part of the patient's experience, although it is not always easy to evaluate the success of the service via clinical outcomes due to the long-term effects, for example. The process of the service would acquire relevance as a source of evaluation in a healthcare context [29] and thus the role of the customer in the service. A widely-used scale in service quality research from customer perception is SERVQUAL, developed by Parasuraman et al. [30], also extended to the health sector in its original or modified version, such as Ameryoun et al. [31]. Other studies propose scales adapted to the context of the study, such as Duggirala et al. [32], where data demonstrated that patient-perceived total quality in the healthcare can be explained by seven dimensions: infrastructure, personnel quality, process of clinical care, administrative process, safety indicators, overall experience of medical care and social responsibility.

The importance of patients in improving health services has encouraged a management approach that places the patient as the critical evaluator of the quality of the health service, focusing on patient experience [33, 34]. McCarthy et al. [34, p. 356] explain that ‘patient experience is formed during the moments when the operation (health service) and consumer (the patient) meet’. Johnston and Kong [33] point out that the customers’ experience is both their personal interpretation of the service process and their role (interactions and involvement) during their journey or flow throughout a series of experience points and the feelings it generates in the customer. The sustainability of the donation system also would depend on positive experiences. The donation process is key to the success of the service, whose ultimate goal is not only for the donor to donate, but to repeat the donation in the future, as suggested by Veerus et al. [35]. It would be necessary to identify the critical aspects of the service donation process that improve the perceived quality of the donor's service and that increase the willingness to donate. Thus, a positive ‘donor experience’ is achieved, creating donor value in a voluntary act and ensuring blood reserves.

To determine the critical aspects of blood donation, it is necessary to know the process that is followed [36], which is succinctly explained in the following paragraphs. When a donor arrives at the donation centre, he/she registers and fills out a short survey about his/her personal history. After waiting in the lobby, they will have an interview to determine their suitability with the donation service staff. Once donors are deemed suitable by blood donation centre’s staff, they enter a waiting room. According to American Red Cross guidelines^1^ on donation process, if donating whole blood, a staff member will clean an area of the donor's arm and insert a new sterile needle for blood collection. It is important to ensure that the donor is comfortable during the act of donation, sitting or lying down. A full blood donation takes between 8 and 10 min. When approximately half a litre of whole blood has been collected, the donation is complete and a staff member will place a bandage on the donor's arm. After donation, donors stay in a recovery room, where they are offered refreshments. Donors leave the centre after the staff make a number of recommendations, e.g., staying at the centre for at least 15 min. Also, Craig et al. [36] outline that total waiting time would be the number of minutes that elapse from the time the donor registers at the reception to the time the needle is inserted, as waiting time deter donating motivation. Literature reveals that there is a large gap in the design and validation of an *ad-hoc* quality scale for blood donation, this is, scales designed specifically for the blood donation context. Exceptions include, Saha and Bhattachrya’s [37] measure service quality provided by blood banks from blood donors’ perspective by an adapted SERVQUAL scale. Martín-Santana and Beerli-Palacio’s [9] also propose an original scale to measure donor experience, including post-donation.

As service quality would be an antecedent of donor loyalty, as Boenigk et al. [38] state, in order to recruit and retain donors, blood donation services should make sure that donors have a satisfactory experience when donating. As suggested by some studies [39, 40], service quality evaluates the service experience quality (cognitive evaluation), which influences overall satisfaction (emotional response to the service experience) and, thus customer loyalty, as suggested by some studies. While the perceived service quality, which is mainly based on informational cues, improves, so does the emotional response to the experience and, consequently, future behavioural intentions. Both constructs -service quality and satisfaction- would contribute to explain customer loyalty. According to Oliver [41, p. 34], ‘loyalty is a deeply held commitment to rebuy or repatronise a preferred product/service consistently in the future, thereby causing repetitive same-brand or same brand-set purchasing, despite situational influences and marketing efforts having the potential to cause switching behaviour’. In blood donation setting, Veerus et al. [35] state that if donors have a poor quality experience the first time they donate, the likelihood of their giving blood again diminishes. However, in the context of the donation process and using an *ad-hoc* quality scale, there are few studies that confirm that relationship, barring some exceptions such as that by Martín-Santana and Beerli-Palacio [9]. Therefore, the following hypothesis (H1) is presented:

1. Service quality of the donation process positively influences donor loyalty.

### Anticipated emotions in the service experience

Several authors suggest that quality evaluation corresponds to a cognitive approach. Gracia et al. [40] think that perceived quality is a cognitive definition, which is based on perceptions or evaluations of discrepancy among expectations and actual service features or levels (such as, tangibles). Service quality would be based on customer cognitions, although they explain that a cognitive approach can predict consumer loyalty, but is not sufficient because it does not take into account the emotional reaction or affective response to the service. All of this might limit the role of quality, from a cognitive point of view, as a predictor of the individual’s behavioural intentions. As a response to the limitations of the cognitive approach, the literature suggests including emotions in models designed to predict consumer behaviour.

Bagozzi et al. [11] define emotions as mental states generated by cognitive evaluations of events or thoughts, which are accompanied by physiological processes, are frequently expressed in physical ways (e.g., facial expressions, postures) and can elicit actions or consequences. As suggested by Michaelidou and Hassan [42], it is perhaps that anticipated emotions (both positive and negative) capture at best one overarching form of emotional response. Based Bagozzi et al. [15], an anticipated emotion is an anticipation of emotional consequences. It is also relevant that anticipated emotions can be studied according to the evaluation of future scenarios where the individual would act, as well as of those scenarios in which the individual would decide not to act [e.g. 15, 43]. Bagozzi et al. [15] explain that both positive anticipated emotions towards action and negative anticipated emotions towards inaction favour the purchase decision-making, and thus literature on anticipated emotions and advertising generally focuses on them. For example, the feeling of guilt or lack of peace-of-mind for not contributing to the donation of life-saving blood would encourage donation. In addition, both negative anticipated emotions toward action and positive anticipated emotions toward inaction negatively influence purchase decisions. For instance, in blood service experience, a positive anticipated emotion of donating may be ‘If I decided to donate, I would feel happy’ and a negative anticipated emotion of non-donating would be ‘If I decide not to donate, I would feel guilty’.

It should be noted that the service literature supports the positive relationship between perceived quality and consumer emotions [16, 44, 45], although it can be inferred from this review that most studies have focused on emotions before and after consumption, but not on anticipated emotions. An exception is the study made by Hur and Jang [46], who found that the perceived healthiness of the advertised foods has a negative influence on anticipated guilt and a positive influence on anticipated pleasure, as healthiness in quick service restaurants is a key quality factor for this sort of restaurant [47].

Based on the above and extrapolating previous results to blood services, the following hypothesis (H2) can be formulated, along with four corresponding sub-hypotheses:

H2. Service quality of the donation process influences anticipated emotions.
  - Service quality of the donation process has a positive influence on positive anticipated emotions of the act of donating.
  - Service quality of the donation process has a negative influence on negative anticipated emotions of the act of donating.
  - Service quality of the donation process has a negative influence on positive anticipated emotions of the act of not donating.
  - Service quality of the donation process has a positive influence on negative anticipated emotions of the act of not donating.

The literature also supports the influence of anticipated emotions in future intentions [e.g. 48]. Schneider et al. [49] provide empirical evidence that positive anticipated emotions (pride) of deciding on pro-environmental options and negative anticipated emotions (guilt) of not deciding on pro-environmental options have a simultaneous effect, albeit with different intensities, on pro-environmental behavioural intentions. Particularly in blood donation, it can be deduced that, although most people experience positive emotions toward donation that drive them to give blood (e.g. pride or self-satisfaction), even overcoming donation barriers (e.g. lack of time, fear of needles), others display negative emotions (e.g., regret or anxiety). Thus, in critical healthcare contexts, although an individual may feel positive emotions about donating (e.g. pride), they may also experience negative emotions (e.g. fear of needles). For instance, Conner et al. [50] indicated that, together with other variables, anticipated negative and positive affective reactions were significant predictors of donation intention. Consequently, both positive emotions (e.g. calmness or relief) and negative emotions (e.g. disappointment at oneself or guilt) caused by the decision not to donate are also very relevant. Jaafar et al. [51] also determined that anticipated regret of non-donation motivates donation. That is, the people who have never donated would be motivated towards donating by a feeling of guilt for not having donated. Romero-Domínguez et al. [52] add that active donors also experience negative emotions, such as general fear and anxiety of donation, which could prevail over the willingness to donate again.

Based on above, therefore, we formulate the following hypothesis (H3) and sub-hypotheses:

H3. Anticipated emotions influence donor loyalty.
  - Positive anticipated emotions of the act of donating have a positive influence on donor loyalty.
  - Negative anticipated emotions of the act of donating have a negative influence on donor loyalty.
  - Positive anticipated emotions of the act of not donating have a negative influence on donor loyalty.
  - Negative anticipated emotions of the act of not donating have a positive influence on donor loyalty.

**Fig. 1:**
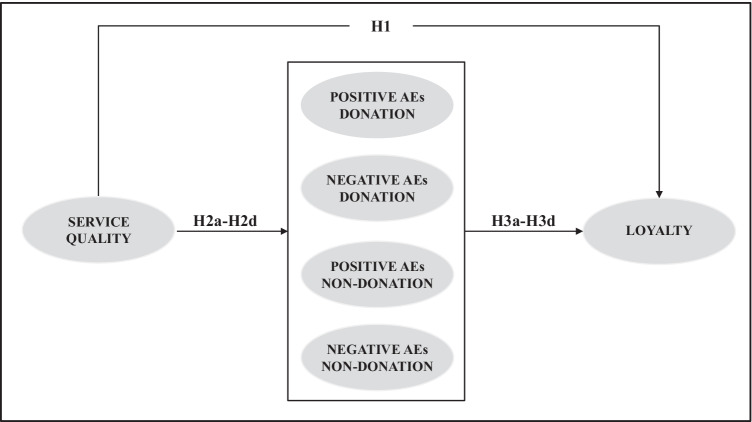
Proposed model

Figure 1 presents the model proposed in this study where the hypotheses formulated are shown, which are the expression of the relationships between quality, anticipated emotions and loyalty in the context of blood donation.

## Materials, methods and data

### Sampling

The study population is composed by active donors, i.e., individuals over 18 years of age live in Spain and have donated blood at least once in the last two years, following the classification proposed by DOMAINE Project [53], which classifies donors into active, inactive and non-donors.

In Spain, blood donation is the responsibility of the so-called transfusion centres, which are ‘health centres where activities are carried out to collect and analyse human blood or their components, regardless of the purpose that they are used for, and to treat, store and distribute them when they are used for transfusion’ [54, p. 31292]. Of the 17 regional blood donation centres in Spain, 14 participated in this study.

Data were collected through an online self-administered questionnaire from March to September 2018. Participating centres sent all their registered donors an e-mail with the URL of the online platform that hosted the questionnaire. This email also explained to them that the research was funded by the Ministry of Economy and Competitiveness and, therefore, endorsed by this Ministry and by the Vice-Rectorate for Research of the authors' university. Also, 24 of Spain's 83 public and private universities also collaborated in this study by distributing the questionnaire by email to all their members (teachers, students, and management and service staff). The donation centres and the universities also used their web and social media to disseminate the questionnaire.

The final sample consisted of 30,621 active donors, 86.9% of who came from the donation centres. The sociodemographic profile of Spanish blood donors, shown in Table 1, is characterised by a balance between men and women, with a wide age distribution; the majority with university studies, workers and a total monthly income between €1,000 and €4,000. The sample distribution according to the donation frequency 2017, shows that almost 40% donated only once compared to 26.8% who donated three or four times. It is necessary to highlight that, in Spain, men are allowed to donate a maximum of four times a year, whereas women are allowed to donate three times a year. This is a measure established for physiological reasons, given that during menstruation women suffer important losses of iron, which must be compensated.

**Table 1:** Sample profile of active Spanish donors

Sociodemographic characteristics	N	%
Sex		
Male	14,464	47.2
Female	16,157	52.8
Age (years)		
18–25	5,440	17.8
26–35	6,186	20.2
36–45	8,337	27.2
> 45	10,658	34.8
Level of education		
No education or primary	3,786	12.4
Secondary	10,972	35.8
University	15,863	51.8
Working		
Yes	23,752	77.6
No	6,869	22.4
Total monthly income (€)		
< 1,000	4,479	14.6
1,001–2,000	12,065	39.4
2,001–4,000	10,932	35.7
> 4,000	3,145	10.3
Annual donation frequency in 2017		
Once	12,222	39.9
Twice	10,198	33.3
3–4 times	8,201	26.8
**Total**	**30,621**	**100.0**

### Measures

#### Service quality

A seven-point Likert scale of 19 items (1-very negative and 7-very positive) was used to assess different aspects related to service quality. In order to propose a valid *ad-hoc* scale relating to donation process quality from the donor’s perspective, some responsible for the donation centres were interviewed and of course, taking into account attributes identified on literature review [35, 55, 56]. The proposed scale consisted of four dimensions: tangibility (3 items), accessibility (4 items), personal attention and professionalism (8 items) and post-donation (4 items). The scale includes aspects related to the physical setting of the service (e.g. ‘The facilities are sufficiently clean’), which have been recognised in the literature on quality measurement in health services, such Duggirala et al. [32], Singh et al. [57], and Swain [58]. Another dimension of the scale assesses the role of the donation service staff (e.g. ‘The staff inspire confidence during the donation’). The role of the staff in the success of the service has also been analysed by Duggirala et al. [32], Pekkaya et al. [59], and Swain [58]. The dimension related to service accessibility measures the donor's opportunity cost in terms of time (e.g. ‘The donation venues' schedule is convenient’), also analysed by Singh et al. [57] and Swain [58]. Another set of variables analyse the donor's experience after donation (e.g. ‘I get a thank you letter or message after each donation’), which has been analysed by Martín-Santana and Beerli-Palacio [9] and Melián-Alzola and Martín-Santana [60].

The empirical analyses carried out helped validate the suitability and goodness-of-fit of the scale, as well as its predictive value (see Section 4.1). The final service quality scale consisted of 17 items (SQ1 to SQ17) after validating their dimensionality through confirmatory factor analyses (CFA). The two items removed from the initial scale were (1) ‘it is easy to park in the centres or donation points’, and (2) ‘the refreshment offered after the donation is fine’. Dimensions of service quality are rated on the donor’s average service experience over all previous episodes.

#### Anticipated emotions (AEs)

A scenario-based question was used. This formula has been used in previous works on AEs [15, 61]. It ‘helps to standardise the social stimulus across respondents and at the same time makes the decision-making situation more real’ [62]. The scenario was defined as follows: ‘Imagine that you are now in front of a mobile blood donation unit and the promoter invites you to donate’. Then, two possible decisions were presented: ‘If you decided NOT TO DONATE…’ and ‘If you decided TO DONATE…’. A series of positive and negative AEs were included for each decision (‘not to donate’ and ‘to donate’), and were measured using 7-point Likert scales (1-strongly disagree and 7-strongly agree), according to the four categories suggested by Bagozzi et al. [15]: 1) positive anticipated emotions of donation (posAEd), 2) negative anticipated emotions of donation (negAEd), 3) positive anticipated emotions of non-donation (posAEnon-d) and 4) negative anticipated emotions of non-donation (negAEnon-d). The decision to measure positive and negative anticipated emotions of donation and non-donation separately was taken because they are two different psychological systems, instead of opposite sides of a single construct [63, 64]. To adapt the scales to this work, we started from the literature on anticipated emotions [15, 63, 65] and on emotions associated to blood donation [e.g. 50]. For instance, in blood donation setting, Williams et al. [21] highlight emotions such as happy, stressed, proud or calm; France et al. [66] outline emotions related to regret and guilt, and Hoogerwerf et al. [67] recognise, among others, anxiety, fear, stress and arousal. Specifically, the three positive anticipated emotions of donation were: happy, proud, and satisfied (AE1, AE2 and AE3). The three negative anticipated emotions of donation were: worried, regretful, and anxious (AE4, AE5 and AE6). The three positive anticipated emotions of non-donation were: relieved, convinced, and calm (eliminated, AE7 and AE8). The three negative anticipated emotions of non-donation were: disappointed, guilty and, angry at oneself (AE9, AE10 and AE11). The item ‘relieved’ was eliminated because the initial CFA demonstrated that its individual reliability was lower than 0.50.

#### Loyalty

A 7-point scale of 4 items (1-totally disagree and 7-totally agree) was used to measure loyalty. This scale included items measuring the two dimensions of loyalty (repetition intention [L1 and L2] and recommendation intention [L3 and L4]), which are considered as measures of future intentions [e.g. 68, 69].

The questionnaire was pretested by 10 collaborators of the donation centres to guarantee their adjustment to the blood donation sector. The questionnaire was also pretested on a sample of 25 active donors. Table 2 shows the final items of each of these scales after validating their dimensionality through CFA.

**Table 2:** Final items of the scales used in this research (Readers may contact the authors to request a copy of the entire questionnaire designed and used by the authors as part of a national research project)

Constructs		Code-Variable	Items
SERVICE QUALITY (SQ)	Tangibility (TANG)	SQ1	The facilities provide privacy during the interview and the donation
		SQ2	The facilities are sufficiently clean
		SQ3	The facilities are cosy and comfortable
	Accessibility (ACCE)	SQ4	The donation venue (either fixed or mobile) is accessible and easily available
		SQ5	The donation venues’ schedule is convenient
		SQ6	Waiting time before blood collection is half an hour at most^*^
		SQ7	The duration of the donation process is convenient
	Personal attention and professionalism (PA&P)	SQ8	The staff perform well
		SQ9	The staff always explain the requisites to donate, the donation procedure and give recommendations for preventing potential negative effects after donation
		SQ10	The staff are friendly and polite
		SQ11	The staff look after my well-being at all times
		SQ12	The staff inspire confidence during the donation
		SQ13	The staff answer my questions accurately
		SQ14	At the end of the donation, the staff showed their gratitude to me
	Post-donation (PD)	SQ15	I get a thank-you letter or message after each donation
		SQ16	The information sent from analysis results is useful
		SQ17	The information that I am sent from analysis results is easy to understand
ANTICIPATED EMOTIONS (AEs)	Positive anticipated emotions of donation (posAEd)	AE1	If I decided TO DONATE, I would feel happy
		AE2	If I decided TO DONATE, I would feel proud
		AE3	If I decided TO DONATE, I would feel satisfied
	Negative anticipated emotions of donation (negAEd)	AE4	If I decided TO DONATE, I would feel worried
		AE5	If I decided TO DONATE, I would regret it
		AE6	If I decided TO DONATE, I would feel anxious
	Positive anticipated emotions of non-donation (posAEnon-d)	AE7	If I decided NOT TO DONATE, I would be satisfied with my decision
		AE8	If I decided NOT TO DONATE, I would feel calm
	Negative anticipated emotions of non-donation (negAEnon-d)	AE9	If I decided NOT TO DONATE, I would feel disappointed
		AE10	If I decided NOT TO DONATE, I would feel guilty
		AE11	If I decided NOT TO DONATE, I would feel angry at myself
LOYALTY	Intention (INT)	L1	I am going to donate blood in the next four months
		L2	I would like to become a regular blood donor (twice or more times a year)
	Recommendation (RECOM)	L3	I encourage my relatives, friends and co-workers to donate blood
		L4	I discuss the positive aspects of blood donation among my relatives, friends and co-workers

^*^ The 10 collaborators of the centres who participated in the questionnaire pretest established this half-an-hour waiting time, and recommended to specify it in the questionnaire. All of them agreed that donors wait, on average, half an hour

## Results

We test the proposed model using structural equation models (SEM). Structural equation modelling is a multivariate statistical analysis technique that consists of the combination of factor analysis and multiple regression analysis, and it is used to analyse the structural relationship between observable or measurable variables (manifest variables or indicators) and latent constructs (hypothesised and unobservable variables). SEM provides a straightforward method of dealing with multiple relationships simultaneously while providing statistical efficiency [70]. The two characteristics that distinguish SEM from other multivariate techniques are (1) estimation of multiple and cross-dependency relationships, and (2) the ability to represent unobserved concepts in these relationships and to take into account measurement error in the estimation process [70].

Before testing the hypotheses, it is necessary to analyse (1) the psychometric properties of the measurement scales using a confirmatory factorial analysis (CFA), that is, the appropriateness of these scales to measure the constructs for which they were designed; (2) the discriminant validity, which is intended to demonstrate the independence between that measurement scales of different constructs of proposed model; and (3) the existence of common method variance (CMV), in order to test the spurious internal consistency. Appendix 1 Table 8 summarises the statistics used in each of these analyses and the interpretation of their values, and the results are shown in Section 4.1. In Section 4.2 we test the proposed model using structural equation models (SEM). Finally, descriptive and multigroup analyses of the model are carried out in Section 4.3.

### Previous statistical analyses to test the proposed model

A second-order confirmatory factorial analysis (CFA) was applied to test the psychometric properties of the measurement scale of service quality, following the procedure established by Hair et al. [70]. It is a technique for interpreting multidimensional scales by bringing various dimensions (Tangibility, Accessibility, Personal attention and professionalism, and Post-donation) under the rubric of a common higher level factor (Service quality). As shown in Table 3, the results of this CFA indicate that this measurement model had achieved a good goodness-fit, since the values of CFI were higher than 0.95 and the values of RMSEA were smaller than 0.08. Most items showed acceptable levels of individual reliability, since the standardised factor loadings were higher than 0.7 and significant at *p* < 0.001. As for the measurements of internal consistency and convergent validity, the values of composite reliability (CR) reached a value exceeding 0.70 and extracted variances (AVE) reached higher than 0.50, except for dimension Accessibility. Cronbach's alphas (α) corroborated the internal consistency of the scale. In short, this scale is valid and reliable. These results confirm that Service quality is a multidimensional construct, with the Post-donation dimension contributing the least to its explanation (0.382). The other three dimensions contribute very positively to the explanation of quality, with loadings higher than 0.7.

**Table 3:** Confirmatory factorial analysis of Service quality

Relationships	Individual reliability			Internal consistency		Convergent validity (AVE)
	Standardised factor loadings	*t*	*p*	Cronbach’s alpha	Composite reliability (CR)	
Fit measures: *χ*^*2*^(115) = 12,762.500, *p* = 0.000, CFI = 0.957, RMSEA = 0.060						
TANG ← **Service quality**	0.774			0.873	0.798	0.515
ACCE ← **Service quality**	0.896	77.716	0.000			
PA&P ← **Service quality**	0.715	8.445	0.000			
PD ← **Service quality**	0.382	53.062	0.000			
SQ1 ← TANG	0.685	124.568	0.000	0.772	0.828	0.618
SQ2 ← TANG	0.787	144.448	0.000			
SQ3 ← TANG	0.875					
SQ4 ← ACCE	0.549	76.526	0.000	0.696	0.725	0.399
SQ5 ← ACCE	0.636	85.464	0.000			
SQ6 ← ACCE	0.623					
SQ7 ← ACCE	0.708	91.522	0.000			
SQ8 ← PA&P	0.773	124.136	0.000	0.917	0.660	0.931
SQ9 ← PA&P	0.687	111.562	0.000			
SQ10 ← PA&P	0.870	137.596	0.000			
SQ11 ← PA&P	0.894	14.747	0.000			
SQ12 ← PA&P	0.905	142.199	0.000			
SQ13 ← PA&P	0.847	134.382	0.000			
SQ14 ← PA&P	0.675					
SQ15 ← PD	0.541	96.181	0.000	0.802	0.828	0.626
SQ16 ← PD	0.881	143.377	0.000			
SQ17 ← PD	0.900					

*TANG* Tangibility; *ACCE* Accessibility; *PA&P* Personal attention and professionalism, and *PD* Post-donation

The scales of anticipated emotions and loyalty were validated together because the low number of loyalty items did not allow for their individual specification. As shown in Table 4, the results of the analyses carried out indicate that both models are valid and reliable.

**Table 4:** Confirmatory factorial analysis of anticipated emotions and loyalty

Relationships	Individual reliability			Internal consistency		Convergent validity (AVE)
	Standardised factor loadings	*t*	*p*	Cronbach’s alpha	Composite reliability (CR)	
Fit measures: *χ*^*2*^(78) = 2,481.378, *p* = 0.000, CFI = 0.990, RMSEA = 0.032						
AE1 ← posAEd	0.896			0.891	0.896	0.743
AE2 ← posAEd	0.853	189.228	0.000			
AE3 ← posAEd	0.835	184.014	0.000			
AE4 ← negAEd	0.845			0.825	0.836	0.632
AE5 ← negAEd	0.839	135.509	0.000			
AE6 ← negAEd	0.691	12.766	0.000			
AE7 ← posAEnon-d	0.825			0.874	0.878	0.783
AE8 ← posAEnon-d	0.941	127.669	0.000			
AE9 ← negAEnon-d	0.827			0.917	0.918	0.789
AE10 ← negAEnon-d	0.922	202.579	0.000			
AE11 ← negAEnon-d	0.913	20.316	0.000			
INT ← **LOYALTY**	0.566			0.667	0.556	0.387
RECOM ← **LOYALTY**	0.673	3.680	0.000			
L1 ← INT	0.505			0.553	0.604	0.445
L2 ← INT	0.797	38.177	0.000			
L3 ← RECOM	0.850			0.852	0.853	0.743
L4 ← RECOM	0.874	91.871	0.000			

*AE1-AE11* items of anticipated emotions; *posAEd* positive anticipated emotions of donation; *negAEd* negative anticipated emotions of donation; *posAEnon-d* positive anticipated emotions of non-donation; *negAEnon-d* negative anticipated emotions of non-donation; *L1-L4* items of loyalty; *INT* Intention, and *RECOM* Recommendation

The discriminant validity of the constructs and their dimensions was tested. To carry out this validation, we created a new variable for each construct and dimension. We did this by means of a weighted average of the scores that respondents assigned to the items/dimensions that made up each dimension/construct, weighted by each of their regression weights in the previous CFA. The results show that the square roots of all AVEs are higher than the elements not on the diagonal, with the exception of five cases (in bold), which refer to correlations between a construct and its dimensions, which is logical (see Table 5). It can be affirmed that the scales also possess discriminant validity.

**Table 5:** Evaluation of the discriminant validity

Constructs	(1)	(2)	(3)	(4)	(5)	(6)	(7)	(8)	(9)	(10)	(11)	(12)
TANG (1)	*0.786*											
ACCE (2)	0.543	*0.632*										
PA&P (3)	0.495	0.521	*0.965*									
PD (4)	0.282	0.274	0.315	*0.791*								
SERVICE QUALITY (5)	**0.814**	**0.827**	**0.731**	0.585	*0.718*							
posAEd (6)	0.139	0.144	0.203	0.116	0.195	*0.862*						
negAEd (7)	-0.011	-0.041	-0.082	-0.001	-0.040	-0.092	*0.795*					
posAEnon-d (8)	-0.014	0.013	-0.029	-0.023	-0.014	-0.222	0.100	0.*885*				
negAEnon-d (9)	0.049	0.042	0.073	0.066	0.072	0.339	0.031	-0.516	0.*888*			
INT (10)	0.185	0.201	0.238	0.131	0.249	0.227	-0.067	-0.094	0.146	0.*667*		
RECOM (11)	0.145	0.138	0.197	0.162	0.206	0.333	0.003	-0.135	0.207	0.280	0.*862*	
LOYALTY (12)	0.197	0.199	0.261	0.184	0.273	0.360	-0.029	-0.147	0.226	**0.677**	**0.896**	0.*622*

(1) Values in italics (on the diagonal) correspond to the square root of the average variance extracted (AVE). Values below the diagonal correspond to the correlations between the constructs/dimensions

(2) *TANG* Tangibility; *ACCE* Accessibility; *PA&P* Personal attention and professionalism, and *PD* Post-donation; *posAEd* positive anticipated emotions of donation; *negAEd* negative anticipated emotions of donation; *posAEnon-d* positive anticipated emotions of non-donation; *negAEnon-d* negative anticipated emotions of non-donation; *INT* Intention, and *RECOM* Recommendation

These results also show that there are positive correlations between the dimensions of service quality (values between 0.274 and 0.543) and between the dimensions of loyalty (0.280). In general terms, it is observed that the dimensions of service quality and the global quality are positively related to the anticipated emotional response motivating donation (positive anticipated emotions of donation and negative anticipated emotions of non-donation) and negatively related to the emotional response that discourages willingness to donate (positive anticipated emotions of non-donation and negative anticipated emotions of donation). The same occurs for loyalty and its two dimensions. However, this level of relationship is higher with positive anticipated emotions of donation.

Three different methods have been used to test the existence of common method variance analysis (CMV) [71]: (1) Harman’s single-factor test; (2) confirmatory factor analysis of Harman’s unique factor and (3) the unmeasured latent method construct (ULMC) technique (see Appendix 1 Table 8). Harman’s single-factor test showed the existence of nine factors, with the first one explaining only 24.2% and the others explaining 47.7% of the variance. These results indicate that CMV does not seem to be a problem in this study, since no method factor emerged. Confirmatory factor analysis of Harman’s unique factor shows the adjustment of the unidimensional model (*χ*^*2*^ = 32,2917.804 with 96 degrees of freedom, CFI = 0.410 and RMSEA = 0.151) is considerably worse than the global measurement model (*χ*^*2*^ = 18,201.879 with 117 degrees of freedom, CFI = 0.967 and RMSEA = 0.036), which suggests that the CMV does not threaten the interpretation of the results. The results of ULMC technique also showed no differences higher than 0.20 between the loadings of the two models, except in some indicators of ‘Personal attention and professionalism’, thus indicating that the CMV bias is not a threat to the data [71]. Therefore, CMV does not seem to be a problem in this study.

### Hypotheses testing with SEM

**Fig. 2:**
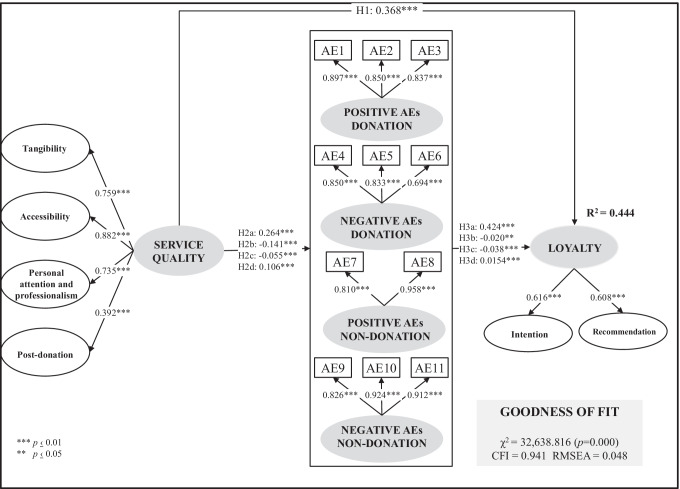
Results of the blood donor loyalty model

A structural equation model (SEM) was used to test the research model following the procedure established by Hair et al. [70]. The variance–covariance matrix was used as input data. The proposed model presented a good goodness-of-fit [*χ*^*2*^(449) = 32,638.816, *p* = 0.000; CFI = 0.941; RMSEA = 0.048], since the CFI value was between 0.90–0.95 and the RMSEA value was lower than 0.08. The results in Fig. 2 allow the following conclusions to be drawn:

- Service quality influences loyalty (*β* = 0.368, *p* = 0.000), thus supporting H1. Thus, the results confirm that the positive experience of the blood donation process increases the donor's willingness to donate, as suggested by Veerus et al. [35]. This finding is also supported by other studies that explain that service quality is an antecedent of loyalty, as demonstrated by the literature review on the antecedents of loyalty by Karunaratna and Kumara [72].
- Service quality influences all anticipated emotions, increasing anticipated emotions motivating donation (posAEd and negAEnon-d) and lowering anticipated emotions motivating non-donation (negAEd and posAEnon-d), and therefore H2a, H2b, H2c and H2d found empirical support. The literature shows a growing interest in the relationship between service quality and consumption emotions [e.g. 73, 74]. Arguello et al. [73] demonstrate that different dimensions of quality of experience influence emotions during service. Hsieh and Yuan [75] also suggest that positive experience perceptions enhance positive emotions. The results obtained in this research also support that positive experience positively influences the emotional expectations motivating donation and negatively influences the emotional expectations motivating no-donating.
- All anticipated emotions influenced loyalty in the way that we had described in the hypotheses. These results were confirmed by the literature, which stated that both categories of anticipated emotions motivating donation (posAEd and negAEnon-d) increase loyalty, whereas both categories of anticipated emotions motivating non-donation (negAEd and posAEnon-d) reduce loyalty. Therefore, H3a, H3b, H3c and H3d found empirical support. These findings confirm that emotional expectations influence consumer behaviour in the direction suggested in the literature [e.g. 15]. These results also support other studies on anticipated emotions on other prosocial behaviours. As Schneider et al. [49] explain that anticipated guilt (negative anticipated emotion of ‘non-action’) promote pro-environmental action, results demonstrate that negAEnon-d (e.g. guilt) would enhance intention to donate.
- The proposed model explained almost 45% of loyalty (*R*^*2*^ = 44.4%). Hair et al. [76] point out that *R*^*2*^ values of 0.75, 0.50 and 0.25 may be considered substantial, moderate and weak, although acceptable *R*^*2*^ are based on the context. Hair et al. [77] explain that *R*^*2*^ values of 0.20 are be considered high in disciplines, such as consumer behaviour, and therefore the R^2^ of loyalty in our research model reaches a relatively high value. As a result, the analysed antecedents of loyalty contribute substantially to explaining future intention to donate blood. Although SEM models do not aim to predict, only to test the model, *R*^*2*^ shows that these variables can explain the variability of loyalty.

### Descriptive and multigroup analyses of the model

The practical implications of this study motivated a descriptive analysis of the three constructs of the model (service quality, anticipated emotions, and loyalty) and its dimensions (see Table 6).

**Table 6:** Descriptive analysis of constructs used (means and standard deviations)

Constructs		Code-Variable	Items/Indicators	*N* = 30,621	
				Mean	SD
SERVICE QUALITY	TANG	SQ1	The facilities provide privacy during the interview and the donation	5.36	1.56
		SQ2	The facilities are sufficiently clean	6.41	0.92
		SQ3	The facilities are cosy and comfortable	6.03	1.15
	ACCE	SQ4	The donation venue (either fixed or mobile) is accessible and easily available	6.29	1.07
		SQ5	The donation venues’ schedule is convenient	5.81	1.40
		SQ6	Waiting time before blood collection is half an hour at most	5.57	1.55
		SQ7	The duration of the donation process is convenient	6.37	0.98
	PA&P	SQ8	The staff perform well	6.63	0.71
		SQ9	The staff always explain the requisites to donate, the donation procedure and give recommendations for preventing potential negative effects after donation	6.52	0.91
		SQ10	The staff are friendly and polite	6.66	0.71
		SQ11	The staff look after my well-being at all times	6.69	0.68
		SQ12	The staff inspire confidence during the donation	6.66	0.70
		SQ13	The staff answer my questions accurately	6.63	0.74
		SQ14	At the end of the donation, the staff showed their gratitude to me	6.34	1.07
	PD	SQ15	I get a thank-you letter or message after each donation	5.56	1.89
		SQ16	The information sent from analysis results is useful	5.77	1.72
		SQ17	The information that I am sent from analysis results is easy to understand	5.80	1.72
TANG				5.96	1.03
ACCE				6.01	0.93
PA&P				6.60	0.65
PD				5.73	1.52
SERVICE QUALITY				6.11	0.73
ANTICIPATED EMOTIONS (AEs)	posAEd	AE1	If I decided TO DONATE, I would feel happy	6.35	1.10
		AE2	If I decided TO DONATE, I would feel proud	6.27	1.24
		AE3	If I decided TO DONATE, I would feel satisfied	6.52	0.96
	negAEd	AE4	If I decided TO DONATE, I would feel worried	1.81	1.54
		AE5	If I decided TO DONATE, I would regret it	1.53	1.36
		AE6	If I decided TO DONATE, I would feel anxious	2.14	1.72
	posAEnon-d	AE7	If I decided NOT TO DONATE, I would be satisfied with my decision	3.90	2.31
		AE8	If I decided NOT TO DONATE, I would feel calm	3.63	2.28
	negAEnon-d	AE9	If I decided NOT TO DONATE, I would feel disappointed	4.72	2.18
		AE10	If I decided NOT TO DONATE, I would feel guilty	4.40	2.21
		AE11	If I decided NOT TO DONATE, I would feel angry at myself	4.17	2.23
posAEd				6.38	1.00
negAEd				1.77	2.64
posAEnon-d				3.76	2.16
negAEnon-d				4.42	2.05
LOYALTY	INT	L1	I am going to donate blood in the next four months	6.21	1.42
		L2	I would like to become a regular blood donor (twice or more times a year)	6.53	1.02
	RECOM	L3	I encourage my relatives, friends and co-workers to donate blood	6.01	1.44
		L4	I discuss the positive aspects of blood donation among my relatives, friends and co-workers	5.93	1.50
INT				6.41	0.98
RECOM				5.97	1.37
**LOYALTY**				6.17	0.97

*SQ1-SQ17* items of service quality; *TANG* Tangibility; *ACCE* Accessibility; *PA&P* Personal attention and professionalism, and *PD* Post-donation; *AE1-AE11* items of anticipated emotions; *posAEd* positive anticipated emotions of donation; *negAEd* negative anticipated emotions of donation; *posAEnon-d* positive anticipated emotions of non-donation; *negAEnon-d* negative anticipated emotions of non-donation; *L1-L4* items of loyalty; *INT* Intention, and *RECOM* Recommendation

The results showed that (1) donors had a good perception of the global service quality (Ms_ervice quality_ = 6.11), with Personal attention and professionalism (M_PA&P_ = 6.60) and Accessibility (M_ACCE_ = 6.01) being the highest rated attributes; (2) it is possible to improve in all quality dimensions, although in the dimension of Personal attention and professionalism, the action scope is rather limited due to the fact that its attributes had very high means and very low standard deviations; (3) the attribute in the dimension of Tangibility, which required more attention, was related to privacy at facilities (M = 5.36, SD = 1.56); (4) the attributes of the dimension of Accessibility, which could be improved, regarded schedule flexibility at donation venues and waiting times before blood collection (SQ5 and SQ6), where deviations are higher than 1; (5) all Personal attention and professionalism attributes had high scores, although staff could show more gratitude to the donor at the end of the donation (SD = 1.07); and (6) there was a relevant action margin in the Post-donation dimension, as all means were below 6 and deviations were high (ranging from 1.72 to 1.89).

The results indicate that (1) among the two types of anticipated emotions motivating donation (posAEd and negAEnon-d), the former showed greater scores (M_posAEd_ = 6.38 and M_negAEnon-d_ = 4.42), with no significant differences in mean values of the items that each type consists of (i.e., between AE1, AE2 and AE3 and between AE9, AE10 and AE11); and (2) among the two types of anticipated emotions motivating non-donation (negAEd and posAEnon-d), the former presented lower scores (M_negAEd_ = 1.77 and M_posAEnon-d_ = 3.76), with some differences in mean values of the items that each type consists of (i.e., between AE4, AE5 and AE6 and between AE7 and AE8).

The results also showed that active donors were very loyal to their donation venues (M = 6.17), with high levels in their intentions to keep donating in the future (M = 6.41) and their willingness to encourage people around them to donate (M = 5.97).

As the literature seems to support that donor experience is a factor affecting donor behaviour [e.g. 78], it might be useful to analyse the existence of significant differences in the mean values of the model constructs as a function of participants' annual donation frequency. A one-way analysis of variance (ANOVA) was used to determine whether the means of the different constructs and their dimensions were similar in the three independent groups, based on the annual donation frequency reported by participant active donors (see Table 7). The results clearly indicate that increased donation frequency leads to a better perception of quality and, of course, for donors to repeat their donation behaviour and to act as prescribers. Regarding emotions, the low values of the *F* statistic, despite its significance, indicate that the differences between the three groups are low, except for the negative anticipated emotions of non-donation (*F* = 15.648, *p* = 0.000). That is, all active donors may feel similar levels of emotions related to happiness, pride and satisfaction (posAEd); worry, regret and anxiety (negAEd), and conviction and calmness (posAEnon-d). However, these levels differ according to donation frequency when it comes to emotions related to disappointment, guilt and anger directed at oneself (negAEnon-d). These emotions are stronger among the most frequent donors.

**Table 7:** One-way ANOVA results: Annual donation frequency

Constructs	Total sample *N* = 30,621		Annual donation frequency						*F*	(*p*)
			Once (*N* = 12,222)		Twice (*N* = 10,198)		3–4 times (*N* = 8,201)			
	Mean	SD	Mean	SD	Mean	SD	Mean	**SD**		
TANG	5.96	1.03	5.89	1.05	5.96	1.01	6.09	0.99	96.434	0.000
ACCE	6.01	0.93	5.90	0.95	6.02	0.92	6.15	0.90	187.503	0.000
PA&P	6.60	0.65	6.56	0.69	6.61	0.64	6.66	0.59	61.680	0.000
PD	5.73	1.52	5.64	1.55	5.72	1.55	5.89	1.43	70.228	0.000
**SERVICE QUALITY**	6.11	0.73	6.03	0.75	6.11	0.72	6.23	0.70	188.668	0.000
**posAEd**	6.38	1.00	6.36	0.99	6.39	0.98	6.39	1.04	3.777	0.023
**negAEd**	1.77	2.64	1.82	2.51	1.73	2.60	1.76	2.86	3.397	0.033
**posAEnon-d**	3.76	2.16	3.76	2.09	3.79	2.15	3.71	2.26	2.881	0.056
**negAEnon-d**	4.42	2.05	4.35	1.99	4.43	2.04	4.51	2.13	15.648	0.000
INT	6.41	0.98	6.01	1.18	6.57	0.78	6.80	0.60	1987.646	0.000
RECOM	5.97	1.37	5.83	1.44	6.04	1.32	6.10	1.31	113.022	0.000
**LOYALTY**	6.17	0.97	5.91	1.08	6.28	0.88	6.41	0.81	795.680	0.000

*TANG* Tangibility; *ACCE* Accessibility; *PA&P* Personal attention and professionalism, and *PD* Post-donation; *posAEd* positive anticipated emotions of donation; *negAEd* negative anticipated emotions of donation; *posAEnon-d* positive anticipated emotions of non-donation; *negAEnon-d* negative anticipated emotions of non-donation; *INT* Intention, and *RECOM* Recommendation

The differences found between active donors according to their annual donation frequency suggest the replication of this model for each of the three groups analysed. The goodness-of-fit results of the model when itis fitted separately for each group are as follows: Once [*χ*^*2*^(111) = 13,351.864, *p* = 0.000; CFI = 0.941; RMSEA = 0.048], Twice [*χ*^*2*^(111) = 11,062.627, *p* = 0.000; CFI = 0.940; RMSEA = 0.048] and 3–4 times [*χ*^*2*^(111) = 9,223.489, *p* = 0.000; CFI = 0.941; RMSEA = 0.049]. These results indicate that the model is stable, since no differences exist between the absolute fit measures (CFI and RMSEA). Logically, the *χ*^*2*^ values change because this statistic is sensitive to sample size, although the significance value remains less than 1%. However, this stability of the model does not imply that annual donation frequency cannot act as a moderating variable and, therefore, affect the direction and/or strength of the relationship between the constructs in the model (Service quality, posAEd, negAEd, posAEnon-d, negAEnon-d and Loyalty).

**Fig. 3:**
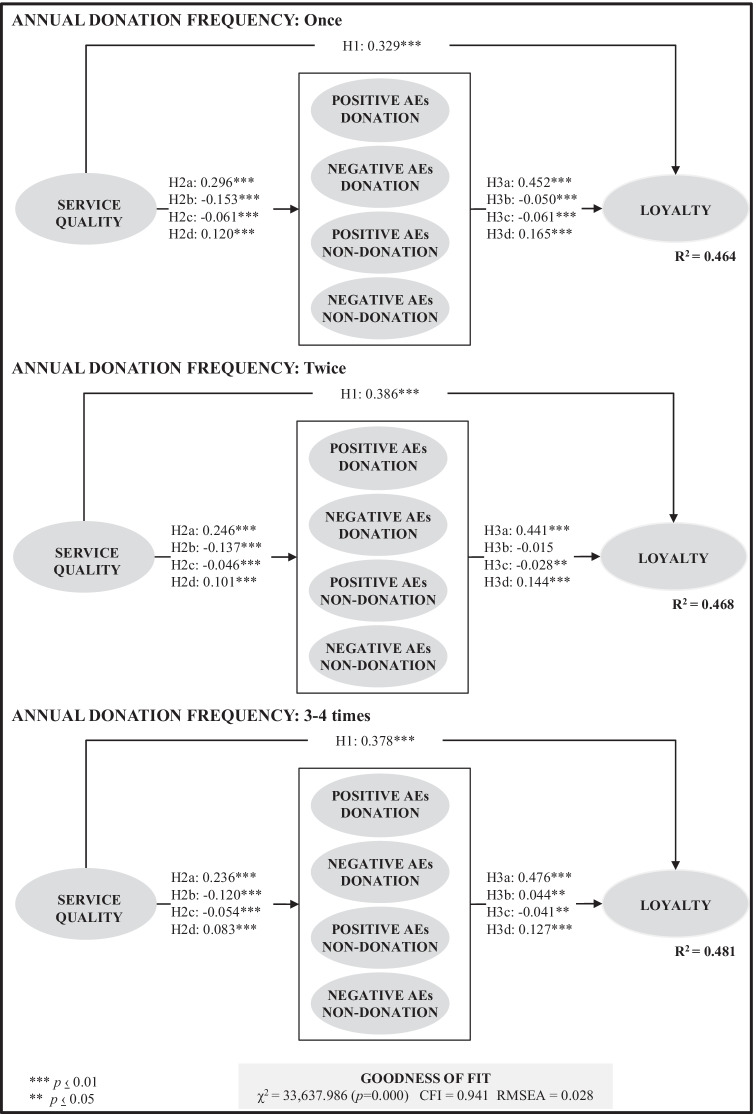
Results of multigroup SEM by annual donation frequency

Figure 3 shows the results of the multigroup model, that is, when the model is adjusted considering the three groups at the same time. As can be seen, the absolute fit measures do not show variations that invalidate the model [CFI = 0.941; RMSEA = 0.028], quite the contrary. This figure also shows the relationships between the different constructs for each of these three groups. Firstly, in all three groups, the proposed model explains more than 45% of the loyalty, where the difference between them amounts to 0.017 percentage points. And, secondly, all hypotheses are supported, with the exception of H3b in the ‘Twice’ group. Regarding the reported pattern of negative anticipated emotions about donating as a function of the donor’s level of experience (H3b), literature provides important insights. Literature also recognises the role of blood donor experience as a factor explaining donor future behaviour [e.g. 78, 6]. Zeithaml et al. [79] also point out that past experience influences service expectations. As a result, it is suggested that the experience of blood donation could modify both the cognitive evaluation (e.g. knowledge of the process, barriers…) and the emotional evaluation (e.g. typology, valence…) of the service. As an example, some authors support that motivations and barriers remain active in experienced donors and may even show a different pattern depending on the degree of experience [e.g. 78, 81]. Literature also supports that the donation process is a prosocial behaviour [22], with emotional implications before, during and after the act of donating, as suggested by Williams et al. [21]. In this regard, diverse studies support that service quality has effects on emotions [e.g. 73]. Moreover, firstly, as motivations can be linked to positive emotions and barriers to negative emotions [e.g. 82], and secondly, motivations and barriers can be modified by experience [e.g. 78], it could be considered that service encounters would allow regulating both positive and negative emotions, as Williams et al. suggest [e.g. 21]. For instance, Mohammed and Essel [78] demonstrate that both for first-time and repeat blood donors, fear of weakness, fear of needless/pain, and fear of contagion are not considered significant barriers to donating blood. Öhrner et al. [82], who analyse motives for cessation and returning to donate blood, also demonstrate that adverse events are of less importance to hinder blood donation.

This result may also be influenced by the fact that in experienced donors, situations or barriers (e.g., needle stick), which could generate negative anticipated emotions, are accepted as part of the service. Martín-Santana and Beerli-Palacio [9] go so far as to say that the act of donating blood requires a process, which in itself, generates fear and aversion. On this basis, we suggest that donors also may declare negative anticipated emotions to donate blood but decide to donate, as they consider such emotions as part of the process of donation. The experience of donating also strengthens the donor's identity (e.g., donation is important) [83], which may balance the anticipated emotions-intention to donate relationship. As an example of this trade-off effect among emotions and intention to donate, Bednall et al. [6] highlight that regret (negative anticipated emotion) is more influential for experienced donors, who would be more aware of the benefits they sacrifice if they miss the opportunity to donate.

As a result, this research highlights the complexity of the donation process, and therefore outlines the need for further research into the moderating role of motivations and perceived barriers of experienced donors in the relationship between anticipated emotions and intention to donate again.

Although not an objective of this study, the moderating role of this variable has led us to analyse the stability of the proposed model in the other five sociodemographic characteristics of the participants (Sex, Age, Level of education, Working and Total monthly income), as well as its possible moderating effect (see Appendices II to VI).

The results of the five one-way also analyses of variance (ANOVAs) show, first, the existence of significant differences in the scores given to all or most of the constructs of the model by the participants depending on sex, age, level of education, working and monthly income. Second, the maximum intra-group dispersion, as measured by standard deviations of means, show in anticipated emotions, mainly in negAEd, posAEnon-d, negAEnon-d. These results indicate that in marketing strategies, donor segmentation based on socio-demographic characteristics may be necessary, since the analysed constructs are affected by sex, age, level of education, working and income level.

Regarding the perception of service quality, the results differ according to the sector (donation, health, tourism, etc.). Thus, in the work of Jain et al. [55], no differences are observed according to sociodemographic characteristics, but other studies [e.g. 84] show results similar to those obtained in this study, for example, that men give lower scores, while older people and those with less education score higher. There are others where the results indicate that young people are more critical in their perception [e.g., 85]. These discrepancies in the effect of sociodemographic characteristics on service quality or loyalty are highlighted in the review work by Vergara Schmalbach et al. (2017) [86]. Some studies collected in this paper show that women and older people score higher on quality and are more loyal, while others show that people with higher income are more loyal, and others show that people with more education score worse on quality.

The studies referring to the differences in the scores of anticipated emotions according to sociodemographic characteristics are very scarce and contradictory [87–89]. This study can be considered as a first attempt at blood donation, whose results should be contrasted with other studies.

The results of the five multigroup models also indicate the absolute fit measures do not show variations that invalidate the proposed model [CFI = 0.941; RMSEA = 0.028], quite the contrary. CFI values range from 0.939 to 0.941, RMSEA values from 0.024 to 0.034 and *R*^*2*^ values from 0.380 to 0.521. However, the intra-group differences in the hypothesis test open new lines of research to explain the moderating role of these sociodemographic variables, as well as other internal donor variables, such as barriers or motivations towards donation. The analysis of sociodemographic characteristics as moderating variables of the relationships of the proposed model in blood donation is supported by the work of Chen, Wu and Guo [19] as the only reference. Their results cannot be directly compared with those of this work because the constructs do not coincide, but these authors do reflect the appropriateness of this analysis. Based on this work, differences in sex and age may be due to the fact that women pay more attention to details of service offered to donors and the environment of blood donation sites, while the older group is more eager to receive warm and thoughtful service and more sensitive to the surrounding environment.

## Discussion

The influence of anticipated emotions in the relationship between service quality and loyalty has been scarcely studied, more particularly in the blood donation process. However, as Martín-Santana et al. [90] state, individuals might anticipate the emotional consequences of their actions, and thus anticipated, this could predict future behaviour. Based on the above, the research model simultaneously analyses the relationships between service quality, anticipated negative and positive emotions (action and non-action) and donor loyalty.

### Academic implications

As for service quality of the donation process, the resulting dimensional structure consisted of four factors: 1) tangibility, 2) accessibility, 3) personal attention, and 4) professionalism and post-donation. Firstly, the different dimensions of the scale are widely regarded as important aspects in the literature [e.g. 8, 37, 55]. Thus, it finds support in the empirically validated scale designed by Martín-Santana and Beerli-Palacio [9]. The data revealed the positive influence of service quality on donor loyalty, with all its dimensions having significant weight. The data also revealed the role anticipated emotions have in predicting donor loyalty, which provided empirical support to the theoretical premises of Williams et al. [21] with regard to the influence of emotions in donor behaviour. In addition, this research contributes to the literature by testing a new model of consumer behaviour that includes anticipated positive and negative emotions, both of action and inaction in blood donation. The analyses confirmed that service quality has a positive effect on the anticipated emotional response motivating donation (positive anticipated emotion of donation and negative anticipated emotion of non-donation) and diminishes the emotional response that discourages willingness to donate (positive anticipated emotion of non-donation and negative anticipated emotion of donation). Thus, we also paid special attention to the suggestions made by Williams et al. [21], who pointed out that the theoretical models applied to blood donation conceived donation as a result of cognitive processes without taking into consideration the role that emotions play in human behaviour.

Findings also demonstrate that negative anticipated emotions of donating do not deter blood donation as donors become more experienced. Regarding the psychology of repeated donors, it is suggested the blood donation be analysed from theoretical frameworks, which may shed light on literature. For instance, from self-determination theory, France et al. [91] note that a positive donation experience contributes to an increased sense of self-efficacy or competence (perceived ability to achieve specific goals), which would enhance intrinsic interest in future donations. Also, diverse studies [e.g. 78] note that some barriers (e.g., fears) could have lesser influence on experienced donors. As a result, and based on above, the moderating role of experienced donors' motivations and barriers in the relationship between anticipated emotions and donor loyalty needs to be studied.

### Managerial implications

As for quality of the donation process, if blood donation centres apply the following positive actions in each dimension of the scale, it is more likely the donor will repeat donation and recommend others to donate.

The ‘tangibility’ dimension includes variables related to privacy in the transaction, cleanliness of the facilities and comfort. Privacy and cleanliness of facilities may affect perceived security or trust in the transaction, so mismanagement of these variables may reduce donor confidence. Thus, Andaleeb and Basu [92] state that well-lit, clean, and ambient facilities contribute to enhance trust and confidence in the donor. In this regard, the cleanliness of the centre can be an indirect indicator of clinical safety in the donation process. Likewise, if the donation process takes approximately 25 min [36], discomfort may increase the perceived inconvenience of your ‘journey’ at the centre and space–time barriers.

The ‘accessibility’ dimension is related to the space–time barriers of the donation process [52, 93]. Because the barriers can diminish the willingness of active donors to donate more often [94], it is suggested that the centre have wide time slots and diversity of locations of fixed and mobile donation centres, where the donors can indicate the day, time and place of donation that best suits their circumstances, as suggested by Martín-Santana et al. [93]. Chen et al. [19] explain that time cost is related to the time spent by a donor on the entire donation process. They state that blood banks should publish blood demand information, collection plans, expected waiting times and recommend donation times in a timely manner, which can effectively reduce the time cost to blood donors.

The ‘personal attention and professionalism’ dimension highlights the role of staff in the success of the process and, above all, in the donor's willingness to repeat the donation. Thus, the staff must be trained both with clinical and psychosocial skills, as Upadhyai et al. [25] recognised. In this regard, Martín-Santana and Beerli-Palacio [9, p.342] highlighted the importance of the selection and proper training of staff: ‘staff are the only ones that can directly and personally transmit to donors their gratitude and also strengthen the social recognition of being a blood donor’. This reveals how significant the staff’s role is, not only in ensuring an optimal service that promotes donor loyalty, but also in having a positive impact on anticipated emotions that motivate people to donate.

The ‘post-donation’ dimension evaluates the last phase of the donation process. This phase is about analysis results and the thank-you letter or message. The results of the analysis are a response to a specific motivation of the donors recognised in the literature as ‘health benefits’ [93], which would favour their future willingness to donate again. The message of gratitude also represents a reward or recognition of the donor's altruistic motivation. With this information, the key aspects of the final phase of the donation process will be designed to positively predispose the donor to future donations.

In addition to the above, in the wake of the COVID-19 (coronavirus disease 2019) pandemic, international health agencies [95] and those with national responsibility [96, 97] have published a series of recommendations and safety protocols for donors, as well as for staff in donation centres. For instance, donors have to inform about their obligation to communicate to their centre if they have symptoms of COVID-19 within 14 days after donation. Also, donors and staff should keep social distance. Consequently, these indications should be included in the design and evaluation of the donation process.

Regarding anticipated emotions, the data reveal that all active donors may feel similar levels of positive anticipated emotions of donating, negative anticipated emotions of donating, and positive anticipated emotions of not donating. However, as the results of the ANOVA analysis indicate (see Table 7), only anticipated negative emotions of not donating differ according to annual donation frequency and increase with donation frequency. Thus, when donors increase the number of donations, they will have more experience and objective information about the donation process, which will increase their negative emotions about not donating. Likewise, as the results reveal that the influence of negative anticipated emotions of donating on donor loyalty depends on the donor's experience (see Fig. 3), a holistic view of donor behaviour including other moderating factors (e.g., motivations and barriers) is required. Given the importance of anticipated emotions in donor loyalty, the donation process should be designed and planned so that, after donation, future expected emotions of donation are as positive as possible. In this regard, Fig. 2 shows that service quality has a positive impact on posAEd and negAEnon-d, which at the same time encourage a greater degree of loyalty.

Furthermore, as suggested by Vichiengior et al. [98], studying the consumer’s anticipations enables organisations to design successful marketing strategies (e.g., verified testimonials) to modify preconceptions about a consumption act. In this regard, social marketing campaigns should design donor recruitment and retention strategies that maximise the benefits of donation, while minimising the perception of barriers and costs. Thus, Martín-Santana et al. [99] propose social marketing actions for non-donors, while Romero-Domínguez et al. [52] address active donors, although there are messages, which can be directed at both types of donor because active donors can also continue to perceive barriers to the act of donating. With regard to the content of the messages of social marketing campaigns, it is essential to strengthen the motivations of the donor. For instance, as Martín-Santana et al. [93] note, marketing campaigns should also focus on the impure or ‘selfish’ dimension of altruism (e.g., personal merit). In addition, Martín-Santana and Beerli-Palacio [9] recommended that repeat donors should have a more active role in donation campaigns, sharing their experiences with other people.

### Limitations and future research

Finally, although this study provides the literature with interesting findings, the results obtained must be validated by similar research done in other geographical areas, in order to compare differences in the role of anticipated emotions on donor behaviour. It is also useful to study the effect other factors (e.g., trust in the blood donation centre or motivations to donate) have on anticipated emotions. In addition, as pointed out by Williams et al. [21], emotions should be studied at all stages of donation. This is particularly relevant in critical situations such as the COVID-19 pandemic. Finally, it would be advisable for centres to carry out a longitudinal follow-up of the constructs included in the model after applying measures to strengthen the impact of quality and anticipated emotions on loyalty. This would support the practical validity of the results obtained from this model.

## Data Availability

The authors undertake to provide the data if requested by any researcher.

## Appendix 1

Table 8

**Table 8:** Previous statistical analyses to test the proposed model

Statistical analysis type	Utility	Statistics and values
Confirmatory factorial analysis (CFA) [70]	To test psychometric properties of the measurement scales. These properties refer to the reliability and validity of the scale. Reliability refers to the consistency while validity refers to the test results' accuracy. Reliability is the degree to which the scale is free from measurement error. In other words, reliability indicates whether one would obtain consistent information if one applied a scale to a population of individuals or groups on repeated occasions. Validity indicates the degree to which a scale is an adequate reflection of the construct to be measured	**GOODNESS OF FIT OF THE MODEL** [80, p. 108]: It indicates how well a specified model reproduces the covariance matrix among the indicator variables. There are alternative GOF measures such as these: - Chi-square statistic: nonsignificant (p > 0.01) but is dependent on sample size - Comparative Fit Index (CFI) ≥ 0.95 - Root Mean Square Error of Approximation (RMSEA) ≤ 0.08 **CONSTRUCT VALIDITY:** It refers to whether a scale measures the construct adequately. In other words, extent to which a set of indicators actually represents the theoretical latent construct those indicators are designed to measure. Several ways are available to estimate the relative amount of construct validity: - Individual item reliability: factor loading with its construct ≥ 0.7 - Composite reliability (internal consistency of the items of a construct) ≥ 0.7 - Convergent validity (the set of items that form this construct represents the same underlying concept): Average Variance Extracted (AVE) [77] ≥ 0.5 - Internal consistency: Cronbach’s alpha coefficient, being 0.7 the minimum acceptable value
Discriminant validity [70]	To demonstrate that measures of constructs that theoretically should not be highly related to each other are, in fact, not found to be highly correlated. This is one of aspects of construct validity. High discriminant validity provides evidence that a construct is unique and captures some phenomena other measures do not	- **Fornell-Larcker** **criterion:** Discriminant validity exists if the correlations between the constructs/dimensions are lower than the square root of the AVE of each of them - **Correlations between construct:** Discriminant validity exists if the correlations between the constructs/dimensions are lower than 1
Existence of common method variance (CMV)	To test the spurious internal consistency that occurs when the apparent correlation among indicators or even constructs is due to their common source	- **Harman’s single-factor test:** To detect the existence of a single factor or several, one of which would explain most of the total variance. If this is so, there is evidence that this problem exists - **Confirmatory factor analysis of Harman’s unique factor:** The adjustment of the unidimensional model must be worse than the proposed measurement model - **The unmeasured latent method construct (ULMC) technique:** This technique consists of adding a first order factor to all measures (indicators) of the measurement model and comparing the factor loadings before and after the inclusion. If there are no differences between the loadings of the two models, this indicates that the CMV bias is not a threat to the data

## Appendix 2

Table 9

**Table 9:** One-way ANOVA results and multigroup SEM by sex

Constructs	Total sample *N* = 30,621		Sex				*t*	*p*
			Male (*N* = 14,464)		Female (*N* = 16,157)			
	Mean	SD	Mean	SD	Mean	SD		
TANG	5.96	1.03	6.00	1.01	5.94	1.04	5.144	0.000
ACCE	6.01	0.93	6.02	0.94	6.00	0.93	2.710	0.007
PA&P	6.60	0.65	6.60	0.63	6.60	0.66	0.530	0.596
PD	5.73	1.52	5.70	1.49	5.76	1.54	3.377	0.001
**SERVICE QUALITY**	6.11	0.73	6.12	0.73	6.10	0.73	2.298	0.022
**posAEd**	6.38	1.00	6.24	1.09	6.49	0.90	22.206	0.000
**negAEd**	1.77	2.64	1.83	2.75	1.72	2.53	3.650	0.000
**posAEnon-d**	3.76	2.16	3.94	2.17	3.59	2.14	14.408	0.000
**negAEnon-d**	4.42	2.05	4.30	2.06	4.52	2.03	9.505	0.000
INT	6.41	0.98	6.46	0.94	6.35	1.02	9.966	0.000
RECOM	5.97	1.37	5.72	1.49	6.19	1.22	30.103	0.000
**LOYALTY**	6.17	0.97	6.06	1.03	6.26	0.91	18.227	0.000

*TANG* Tangibility; *ACCE* Accessibility; *PA&P* Personal attention and professionalism, and *PD* Post-donation; *posAEd* positive anticipated emotions of donation; *negAEd* negative anticipated emotions of donation; *posAEnon-d* positive anticipated emotions of non-donation; *negAEnon-d* negative anticipated emotions of non-donation; *INT* Intention, and *RECOM* Recommendation

## Appendix 3

Table 10

**Table 10:** One-way ANOVA results and multigroup SEM by age

Constructs	Total sample *N* = 30,621		Age								*F*	*p*
			18–25 (*N* = 5,440)		26–35 (*N* = 6,186)		36–45 (*n* = 8,337)		> 45 (*n* = 10,658)			
	Mean	SD	Mean	SD	Mean	SD	Mean	SD	Mean	SD		
TANG	5.96	1.03	5.91	1.03	5.97	1.02	5.95	1.04	6.00	1.01	9.936	0.000
ACCE	6.01	0.93	5.84	0.95	5.91	0.97	6.00	0.94	6.16	0.87	173.326	0.000
PA&P	6.60	0.65	6.49	0.69	6.55	0.70	6.62	0.64	6.67	0.59	105.918	0.000
PD	5.73	1.52	5.69	1.50	5.67	1.56	5.70	1.54	5.81	1.49	16.014	0.000
**SERVICE QUALITY**	6.11	0.73	6.01	0.74	6.06	0.74	6.11	0.74	6.20	0.70	97.889	0.000
**posAEd**	6.38	1.00	6.44	0.88	6.45	0.93	6.37	1.01	6.30	1.08	37.559	0.000
**negAEd**	1.77	2.64	1.85	2.30	1.75	2.59	1.74	2.69	1.77	2.78	1.952	0.119
**posAEnon-d**	3.76	2.16	3.45	1.92	3.55	2.06	3.85	2.19	3.95	2.28	89.829	0.000
**negAEnon-d**	4.42	2.05	4.47	1.88	4.51	1.98	4.39	2.08	4.37	2.13	7.792	0.000
INT	6.41	0.98	6.36	0.98	6.35	1.01	6.39	0.98	6.47	0.97	27.834	0.000
RECOM	5.97	1.37	6.15	1.23	6.17	1.24	5.92	1.40	5.80	1.46	134.278	0.000
**LOYALTY**	6.17	0.97	6.25	0.91	6.25	0.93	6.13	0.98	6.11	1.01	43.812	0.000

*TANG* Tangibility; *ACCE* Accessibility; *PA&P* Personal attention and professionalism, and *PD* Post-donation; *posAEd* positive anticipated emotions of donation; *negAEd* negative anticipated emotions of donation; *posAEnon-d* positive anticipated emotions of non-donation; *negAEnon-d* negative anticipated emotions of non-donation; *INT* Intention, and *RECOM* Recommendation

## Appendix 4

Table 11

**Table 11:** One-way ANOVA results and multigroup SEM by level of education

Constructs	Total sample *N* = 30,621		Level of education						*F*	*p*
			No education or primary (*N* = 3,786)		Secondary (*N* = 10,972)		University (*n* = 15,863)			
	Mean	SD	Mean	SD	Mean	SD	Mean	SD		
TANG	5.96	1.03	6.10	1.01	5.97	1.02	5.93	1.03	43.403	0.000
ACCE	6.01	0.93	6.17	0.90	6.01	0.93	5.97	0.94	68.753	0.000
PA&P	6.60	0.65	6.68	0.65	6.60	0.64	6.58	0.65	37.560	0.000
PD	5.73	1.52	5.87	1.58	5.75	1.54	5.69	1.49	21.988	0.000
**SERVICE QUALITY**	6.11	0.73	6.24	0.73	6.12	0.73	6.08	0.73	76.835	0.000
**posAEd**	6.38	1.00	6.56	0.95	6.42	0.98	6.30	1.02	119.791	0.000
**negAEd**	1.77	2.64	2.20	3.58	1.80	2.70	1.65	2.29	67.692	0.000
**posAEnon-d**	3.76	2.16	3.69	2.31	3.68	2.16	3.83	2.11	17.332	0.000
**negAEnon-d**	4.42	2.05	4.64	2.19	4.51	2.05	4.31	2.00	57.329	0.000
INT	6.41	0.98	6.54	0.91	6.45	0.94	6.34	1.03	78.266	0.000
RECOM	5.97	1.37	6.24	1.22	6.03	1.32	5.86	1.43	136.289	0.000
**LOYALTY**	6.17	0.97	6.38	0.88	6.22	0.94	6.08	1.01	169.935	0.000

*TANG* Tangibility; *ACCE* Accessibility; *PA&P* Personal attention and professionalism, and *PD* Post-donation; *posAEd* positive anticipated emotions of donation; *negAEd* negative anticipated emotions of donation; *posAEnon-d* positive anticipated emotions of non-donation; *negAEnon-d* negative anticipated emotions of non-donation; *INT* Intention, and *RECOM* Recommendation

## Appendix 5

Table 12

**Table 12:** One-way ANOVA results and multigroup SEM by working

Constructs	Total sample *N* = 30,621		Working				*t*	*p*
			Yes (*N* = 23,752)		No (*N* = 6,869)			
	Mean	SD	Mean	SD	Mean	SD		
TANG	5.96	1.03	5.96	1.02	5.97	1.03	0.532	0.595
ACCE	6.01	0.93	6.01	0.94	6.00	0.91	1.374	0.170
PA&P	6.60	0.65	6.61	0.64	6.57	0.65	4.790	0.000
PD	5.73	1.52	5.73	1.52	5.76	1.51	1.551	0.121
**SERVICE QUALITY**	6.11	0.73	6.11	0.73	6.10	0.72	1.012	0.311
**posAEd**	6.38	1.00	6.36	1.02	6.43	0.94	5.464	0.000
**negAEd**	1.77	2.64	1.74	2.63	1.87	2.65	3.584	0.000
**posAEnon-d**	3.76	2.16	3.80	2.17	3.60	2.11	6.634	0.000
**negAEnon-d**	4.42	2.05	4.40	2.05	4.48	2.02	2.779	0.005
INT	6.41	0.98	6.42	0.98	6.37	1.01	3.660	0.000
RECOM	5.97	1.37	5.94	1.39	6.09	1.29	8.232	0.000
**LOYALTY**	6.17	0.97	6.16	0.98	6.22	0.95	4.609	0.000

*TANG* Tangibility; *ACCE* Accessibility; *PA&P* Personal attention and professionalism, and *PD* Post-donation; *posAEd* positive anticipated emotions of donation; *negAEd* negative anticipated emotions of donation; *posAEnon-d* positive anticipated emotions of non-donation; *negAEnon-d* negative anticipated emotions of non-donation; *INT* Intention, and *RECOM* Recommendation

## Appendix 6

Table 13

**Table 13:** One-way ANOVA results and multigroup SEM by total monthly income (€)

Constructs	Total sample *N* = 30,621		Total monthly income (€)								*F*	*p*
			< 1,000 (*N* = 4,479)		1,001–2,000 (*N* = 12,065)		2,001–4,000 (*n* = 10,932)		> 4,000 (*n* = 3,145)			
	Mean	SD	Mean	SD	Mean	SD	Mean	SD	Mean	SD		
TANG	5.96	1.03	5.98	1.08	5.97	1.02	5.95	1.01	5.95	1.02	0.921	0.430
ACCE	6.01	0.93	5.96	0.98	6.00	0.93	6.03	0.92	6.05	0.91	8.258	0.000
PA&P	6.60	0.65	6.57	0.72	6.60	0.64	6.61	0.64	6.62	0.59	5.093	0.002
PD	5.73	1.52	5.75	1.57	5.75	1.52	5.72	1.49	5.65	1.53	4.001	0.007
**SERVICE QUALITY**	6.11	0.73	6.09	0.78	6.11	0.73	6.12	0.71	6.11	0.70	0.984	0.399
**posAEd**	6.38	1.00	6.47	0.98	6.41	0.97	6.34	1.02	6.25	1.07	38.703	0.000
**negAEd**	1.77	2.64	2.04	3.15	1.85	2.77	1.61	2.31	1.65	2.32	35.123	0.000
**posAEnon-d**	3.76	2.16	3.59	2.21	3.70	2.14	3.85	2.15	3.85	2.17	20.671	0.000
**negAEnon-d**	4.42	2.05	4.51	2.11	4.47	2.03	4.35	2.03	4.34	2.05	11.652	0.000
INT	6.41	0.98	6.39	1.02	6.41	0.97	6.40	0.98	6.42	0.99	0.594	0.619
RECOM	5.97	1.37	6.20	1.27	6.05	1.31	5.85	1.43	5.75	1.47	109.688	0.000
**LOYALTY**	6.17	0.97	6.29	0.95	6.21	0.94	6.10	0.99	6.06	1.01	60.999	0.000

*TANG* Tangibility; *ACCE* Accessibility; *PA&P* Personal attention and professionalism, and *PD* Post-donation; *posAEd* positive anticipated emotions of donation; *negAEd* negative anticipated emotions of donation; *posAEnon-d* positive anticipated emotions of non-donation; *negAEnon-d* negative anticipated emotions of non-donation; *INT* Intention, and *RECOM* Recommendation

## Footnotes

1 https://www.redcrossblood.org/donate-blood/blood-donation-process/donation-process-overview.html
